# A Rational Combination of *Cyclocarya paliurus* Triterpene Acid Complex (TAC) and Se-Methylselenocysteine (MSC) Improves Glucose and Lipid Metabolism via the PI3K/Akt/GSK3β Pathway

**DOI:** 10.3390/molecules28145499

**Published:** 2023-07-19

**Authors:** Xichen Bai, Hong Zhou, Dan Luo, Dan Chen, Jianyuan Fan, Xiaoting Shao, Jun Zhou, Wei Liu

**Affiliations:** 1 College of Life Science and Technology, Huazhong University of Science and Technology, Wuhan 430074, Chinahz199268@hust.edu.cn (H.Z.); 2 Wuhan Bestcarrier Biotechnology Co., Ltd., Wuhan 430075, China; 3 Enshi Savant Ecological Agriculture Development Co., Ltd., Enshi 445099, China; 4 Hubei Key Laboratory of Bioinorganic Chemistry & Materia Medica, Hubei Engineering Research Center for Biomaterials and Medical Protective Materials, Key Laboratory of Material Chemistry for Energy Conversion and Storage, Ministry of Education, School of Chemistry and Chemical Engineering, Huazhong University of Science and Technology, Wuhan 430074, China; 5 National Engineering Research Center for Nanomedicine, Huazhong University of Science and Technology, Wuhan 430075, China

**Keywords:** *Cyclocarya paliurus* triterpenic acid, Se-methylselenocysteine, glycolipid metabolism, PI3K/AKT/GSK3β pathway, oxidative stress

## Abstract

*Cyclocarya paliurus* (CP) contains triterpene acids that can improve glucose and lipid metabolism disorders. However, controlling the composition and content of these active ingredients in CP extracts is challenging. The main active components in CP triterpene acids, including ursolic acid (UA), oleanolic acid (OA), and betulinic acid (BA), exhibit antihyperglycemic and antihypertensive effects. The response surface methodology was utilized to design and optimize the ratio of UA, OA, and BA based on the inhibition rate of pancrelipase and α-amylase. The proportional mixture of UA, OA, and BA resulted in the formation of a complex known as *Cyclocarya paliurus* triterpenoid acid (TAC). Se-methylselenocysteine (MSC), a compound with various physiological functions such as antioxidant properties and tumor inhibition, has been used in combination with TAC to form the TAC/MSC complex. Our data demonstrate that TAC/MSC improved palmitic acid (PA)-induced insulin resistance in HepG2 cells through activating the phosphoinositide 3-kinase (PI3K) /protein kinase B (AKT)/glycogen synthase kinase 3 beta (GSK3β) pathway. Moreover, TAC/MSC effectively improved hyperglycemia, glucose intolerance, insulin resistance, and lipid metabolism disorder in mice with type 2 diabetes mellitus (T2DM), attenuated hepatic steatosis, and reduced oxidative stress to alleviate T2DM characteristics.

## 1. Introduction

*Cyclocarya paliurus* (Batalin) Iljinsk (CP), a plant of Juglandaceae, is a native species in China and is widely distributed in the mountainous regions of Hubei, Hunan, Jiangxi, Guangxi, Guizhou, and other provinces in southern China, at altitudes of 400–2000 m [1]. CP is also known as sweet tea tree because of its unique sweet taste. Local and medical communities generally use CP leaves as functional foods or therapeutic agents [1,2]. *CP* leaves contain numerous active ingredients, including polysaccharides, triterpenoids, flavonoids, phenolic acids, and saponins [3,4,5]. Many studies have found that *CP*-derived triterpenoids possess various bioactivities, such as hypoglycemic, hypolipidemic, anti-inflammatory, and antioxidative stress effects [6,7,8,9]. Zhao et al. investigated the effect of *Cyclocarya paliurus* triterpene-acid-enriched fraction (CPT) on non-alcoholic fatty liver disease (NAFLD). The results showed that CPT (100 mg/kg) could protect mitochondrial function and improve oxidative stress by activating nuclear factor erythroid 2-related factor 2 (Nrf2). In terms of hypoglycemic effect and improvement in insulin resistance, Zheng et al. [9] investigated the effects of CPT on high-fat-diet-induced insulin resistance in C57BL/6 mice. The results showed that CPT (160 mg/kg) significantly improved insulin resistance induced by high-fat diet in mice and reduced blood glucose, serum triacylglycerol (TG), total cholesterol (TC), and liver TG and TC levels. Wu et al. evaluated the antihyperlipidemic potential of the triterpenic-acid-enriched fraction (TAE) from *C. paliurus*. The results showed that TAE administration (400 mg/kg) reduced arterial lipid levels in serum and liver, in addition to TAE treatment (200 and 400 mg/kg), and inhibited TNF-α expression by 36.2 and 56.2%, respectively.

However, the content and yield of triterpenoids in the leaves of *CP* vary greatly due to differences in harvesting season, growing region, and the extraction methods used by researchers, which in turn affect the biological activity of CP. Liu et al. [8] studied the content of triterpenes in ethanol and water extracts of CP from Jinzhongshan, Muchuan, Wufeng, Anji, and Suining and studied the effect of ethanol extracts on blood lipid levels in streptozotocin (STZ)-induced diabetes mice. The results showed that the total triterpenoid content in the ethanol extracts of the above five regions was 5.69, 4.39, 8.15, 6.70, and 7.70 mg/g, respectively, and the total triterpenoid content in the water extracts was 0.08, 0.15, 0.13, 0.05, and 0.07 mg/g, respectively. Numerous compounds have been isolated and identified from CP triterpenoid acids (TAs), with ursolic acid (UA), oleanolic acid (OA), and betulinic acid (BA) serving as representative triterpenoid components [10,11]. Wu et al. [7] demonstrated that the ethanolic extract of CP possesses antihyperlipidemic activity attributed to its abundant content of these three pentacyclic triterpenes mentioned above. Recent research has also suggested that OA, UA, and BA, as well as plant extracts rich in these TAs, could be utilized as dietary supplements or pharmaceutical agents to improve glucose and lipid metabolism [12].

Selenium (Se) is an essential element that maintains the health of mammals. However, there is a universal problem that the soil in over 50% of regions in our country lacks Se [13]. The enrichment of natural Se resources in a few Se-rich regions cannot meet the demand for Se supplements in healthy populations. Therefore, people use a series of biofortification strategies, such as plant bioaugmentation, to develop Se-rich foods [14]. In our previous studies, organic Se fertilizer was manufactured to obtain Se-rich *CP* leaves that could meet the national Se-rich tea standard (0.25~4.00 mg/kg) [15]. We also found that the oral administration of Se-CPP (0.6 g/kg) to diabetic mice induced by a high-fat diet and intraperitoneal injection of streptozotocin increased serum glutathione peroxidase (GSH-Px) and superoxide dismutase (SOD) activity in mice. Se-CPP (0.6 g/kg) also showed better hypoglycemic effects compared with those containing the same dose of CPP + sodium selenite (CPP 0.6 g/(kg·d), Na_2_SeO_3_ 4.34 μg/(kg·d)). The same study also found no adverse effect of Se-CPP on blood glucose in normal mice [16]. Currently, the Se forms approved for nutritional food fortifiers contain sodium selenite, sodium selenate, selenoprotein, Se-enriched edible fungus powder, Se-methylselenocysteine (MSC), selenized carrageenan, and Se yeast [14]. MSC is a major Se compound in Se-rich plants such as garlic and broccoli florets. Recent studies on MSC have mainly focused on its antitumor and antioxidant activity [17], as well as its potential to prevent Alzheimer’s disease [18]. The study by Hu et al. [19] showed that selenocysteine and MSC are more easily absorbed by the gastrointestinal tract than selenomethionine, the main form of organic selenium present in selenium-rich edible mushrooms and selenium-rich yeast, with absorption rates of 90%, 76%, and 51%, respectively. Moreover, MSC supplementation has been shown to be beneficial for diabetes therapy due to an improvement in both hyperglycemia and renal function in alloxan-induced type 1 diabetic rabbits [20].

Previously, we successfully confirmed that Se enrichment can increase the content of bioactive substances in *CP* leaves [15], and form new Se polysaccharide compounds with improved activity. Meanwhile, it has also been verified that the combination of *CP* polysaccharide and sodium selenite could increase hypoglycemic activity. As a food additive approved by the National Health Commission (NHC, Beijing, China) and Food and Drug Administration (FDA, Silver Spring, MD, USA), MSC has better bioavailability than inorganic Se and is easier to be absorbed by the gastrointestinal tract [19,21], which makes it more conducive as a food additive in *CP* health food. To date, studies on the efficacy of *CP* are mostly related to ethanol or water extracts, and there is no report on whether MSC can promote the hypoglycemic effect of *CP* as a food additive. The composition and content of the active ingredients contained in the triterpenic acid extract of *CP* are difficult to control, which restricts its practical application in the fields of medicine and health food. In this study, the representative triterpenic acids of *CP*, ursolic acid, oleanolic acid, and betulinic acid, were selected, and the best compounding ratio was determined through compounding studies to obtain the *Cyclocarya paliurus* triterpene acid complex (TAC), and this was compounded with MSC to obtain a triterpenic acid/Se-methylselenocysteine (TAC/MSC) complex of *CP* to provide a theoretical and experimental basis for the research and development of new selenium-rich health foods.

## 2. Results

### 2.1. Response Surface Analysis and Optimization

**Figure 1:**
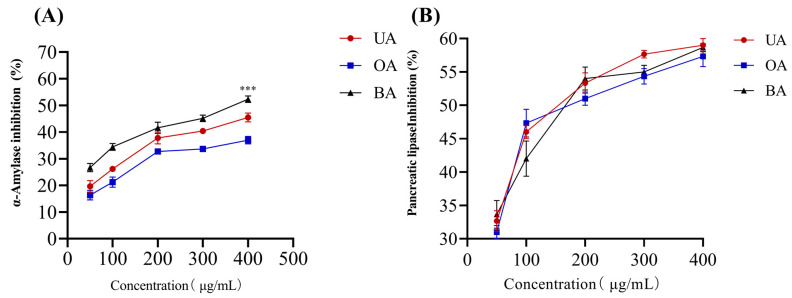
Inhibitory activity of different concentrations of UA, BA, and OA against α-amylase and pancrelipase. (**A**) α-amylase inhibition activity of UA, OA, and BA; (**B**) pancreatic lipase inhibition activity of UA, OA, and BA. These data are presented as the mean ± SD (n = 3). *** *p* < 0.001, compared to the OA (400 μg/mL).

The influence of UA, OA, and BA at different concentrations on the activities of α-amylase and pancrelipase is depicted in Figure 1. As shown in Figure 1, UA, OA, and BA showed a dose-dependent inhibition of a-amylase and pancrelipase. BA exhibited significantly higher inhibitory activity on α-amylase compared to UA and OA, and no substantial increase was observed at concentrations above 200 μg/mL. The impact on pancrelipase is shown in Figure 1B; there was no significant difference in the inhibitory effect of UA, OA, and BA on pancreatic lipase. At a concentration of 200 μg/mL, BA, OA, and UA showed 53, 51, and 54% pancrelipase inhibition, respectively, and no substantial increase was observed at concentrations above 200 μg/mL. Based on the above results, concentrations of UA, OA, and BA ranging from 100 to 400 μg/mL were selected as the single factor range.

In the statistical analysis of Box–Behnken design (BBD), 15 experimental conditions were selected and examined, including three replicated center points. Based on the single factor experiment, a Box–Behnken design of the central combination experiment was adopted, and three factors of UA, BA and OA were selected. The inhibition (%) of α-amylase and pancreatic lipase was of two levels. The effects of the independent factors (the contents of UA, BA, and OA) on the inhibition (%) of α-amylase and pancreatic lipase are shown in Table 1.

Taking the pancreatic lipase inhibition (%) as the response value, the RSM regression model analysis and the variance analysis of pancreatic lipase inhibition (%) of the various compounds are shown in Table 2. The p value is 0.0532, which is larger than 0.05, indicating that the response surface regression model has a good fit, and therefore can be used to optimize the formulation of the compound. The above results show that the influence of various factors on pancreatic lipase inhibition (%) in the compound was in the order of A > B > C. Therefore, UA exhibited the strongest inhibitory effect on pancreatic lipase activity, BA was the second, and OA was the smallest.

Regarding α-amylase inhibition (%) as the response value, regression model analysis and variance analysis with α-amylase inhibition (%) are shown in Table 3. The results show that the influence of various factors on α-amylase inhibition (%) in the compound was in the order of B > A > C, that is, BA exhibited the strongest inhibitory effect on α-amylase, UA was the second, and OA was the smallest.

The optimal values were found to be 348 μg/L for the content of UA, 354 μg/L for the content of BA, and 398 μg/L for the content of OA, which resulted in a maximum inhibition of α-amylase of 63.9% and pancreatic lipase of 67.2%, with a desirability value of 1.00.

### 2.2. TAC/MSC-Enhanced Glucose Consumption in HepG2 Cells

**Figure 2:**
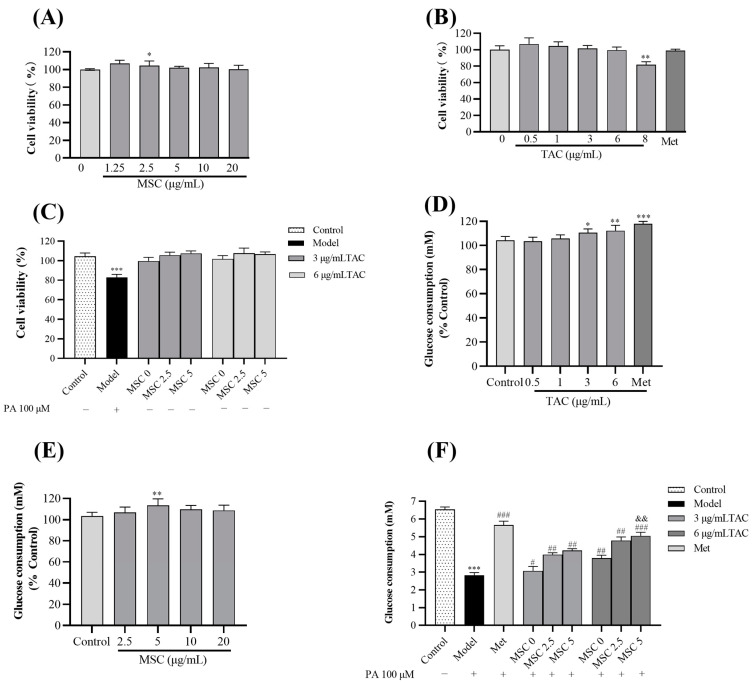
Effect of TAC/MSC on glucose consumption in HepG2 cells. HepG2 cells were treated with MSC, TAC, TAC/MSC (TAC 3, 6 μg/mL; MSC 2.5, 5 μg/mL (13.7, 27.4 μM)), Metformin (Met) (10 μg/mL), or PA (100 μmol/L) for 24 h. The MTT assay was used to determine cell viability. (**A**) Cell viability after exposure to MSC; (**B**) cell viability after exposure to TAC or Metformin; (**C**) cell viability after exposure to TAC/MSC or PA. Cells in the model group were treated with PA only. (**D**) Glucose consumption after exposure to TAC. (**E**) Glucose consumption after exposure to MSC. (**F**) HepG2 cells were treated with or without 0.1 mM PA and TAC/MSC for 24 h. Cells in the model group were treated with PA only. These data are presented as the mean ± SD (n = 5). Vertical lines represent the standard deviations of five replicates. * *p* < 0.05, ** *p* < 0.01, *** *p* < 0.01, compared to the control. ^#^ *p* < 0.05, ^##^ *p* < 0.01, ^###^ *p* < 0.001, compared to the model. ^&&^ *p* < 0.01, compared to the 6 μg/mL TAC group. Metformin (10 μg/mL) was used as a positive control.

As shown in Figure 2A–C, no obvious inhibitory effect on cell growth was observed in treatment with Se-methylselenocysteine (1.25~20 μg/mL), TAC (0.25~6 μg/mL), and TAC (3, 6 μg/mL)/MSC (2.5, 5 μg/mL). According to Figure 2D,E, TAC (3, 6 μg/mL) and MSC (5 μg/mL) could significantly increase glucose consumption in HepG2 cells without palmitic acid (PA), while 2.5 μg/mL and 10 μg/mL MSC had no significant difference in glucose consumption. However, since 10 μg/mL MSC exceeded the standard for Se-rich tea, 2.5 and 5 μg/mL were utilized as the subsequent experimental concentrations of MSC. The glucose consumption of HepG2 cells was significantly reduced after PA treatment (*p* < 0.001), while TAC administration improved this situation. Interestingly, after adding MSC (5 μg/mL), TAC (6 μg/mL) significantly increased the glucose consumption of insulin-resistant cells (*p* < 0.01) (Figure 1F).

### 2.3. TAC/MSC Can Increase Glycogen Synthesis in Insulin-Resistant HepG2 Cells

**Figure 3:**
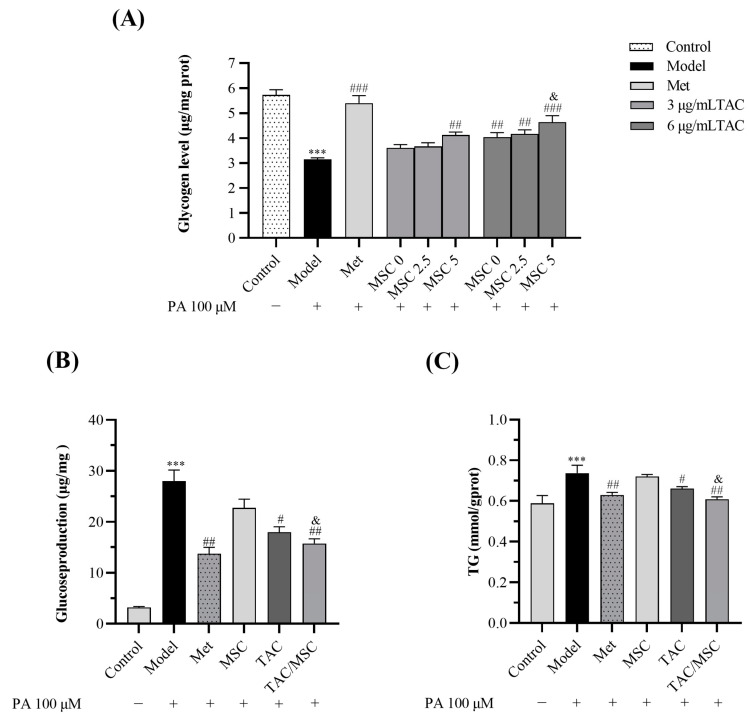
TAC/MSC treatment increased the glycogen content and decreased glucose production and TG content in PA-treated HepG2 cells. Cells in all groups except the control group were treated with PA (0.1 mM). TAC (6 μg/mL), MSC (5 μg/mL (27.4 μM)), or metformin (10 μg/mL) were used in the glucose production assay and triglyceride content assay. Cells in the model group were treated with PA only. (**A**) The intracellular glycogen content. (**B**) Glucose production in culture medium (DMEM without glucose). (**C**) The intracellular TG content. These data are presented as the mean ± SD (n = 3). *** *p* < 0.01, compared to the control. ^#^ *p* < 0.05, ^##^ *p* < 0.01, ^###^ *p* < 0.001, compared to the model. ^&^ *p* < 0.05, compared to the 6 μg/mL TAC group.

PA treatment can inhibit glycogen synthesis and activate gluconeogenesis in HepG2 cells [22]. As shown in Figure 3A, exposing HepG2 cells to 0.1 mM PA for 24 h (model group) remarkably reduced the glycogen content in HepG2 cells. TAC or TAC/MSC treatment led to considerably increased intracellular glycogen content. In addition, PA-enhanced gluconeogenesis was reduced by TAC (6 μg/mL) and TAC (6 μg/mL)/MSC (5 μg/mL) treatment (Figure 3B). Similarly to the results of the glucose consumption experiment, MSC (5 μg/mL) promoted the effects of TAC (6 μg/mL) on glycogen synthesis and gluconeogenesis (*p* < 0.05, Figure 3A,B). To determine whether MSC can promote TAC to reduce lipid accumulation in HepG2 cells exposed to PA, the intracellular triglyceride (TG) content was determined. Compared with the control group, the model group showed a significant increase in TG content (*p* < 0.001) (Figure 3C). The co-administration of MSC and TAC can significantly decrease intracellular TG content.

### 2.4. Effects of TAC/MSC on P-PI3K, PI3K, P-AKT, AKT, P-GSK3β, and GSK3β in HepG2 Cells

**Figure 4:**
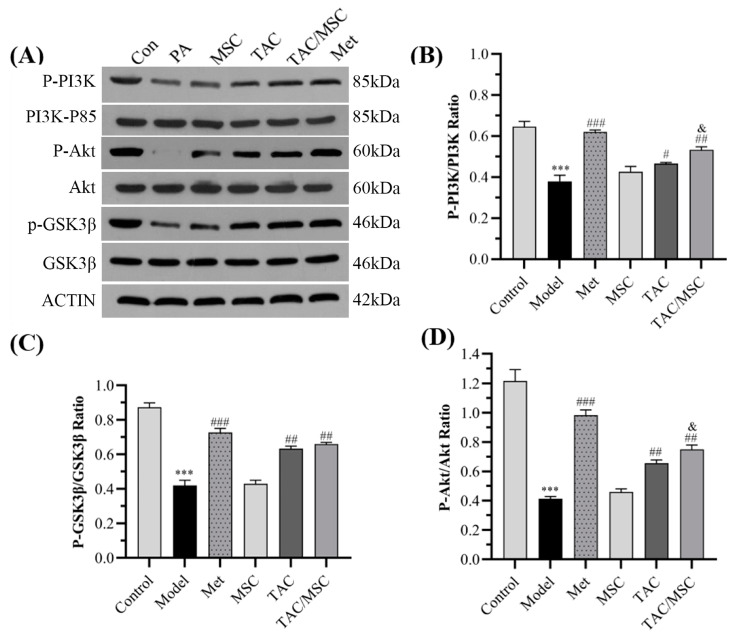
Effect of TAC/MSC on phosphoinositide 3-kinase (PI3K)/P-protein kinase B (AKT)/glycogen synthase kinase 3 beta (GSK3β) signaling pathway in PA-treated HepG2 cells. Cells in all groups except the control group were treated with PA (0.1 mM). TAC (6 μg/mL), MSC (5 μg/mL (27.4 μM)), or metformin (10 μg/mL) were used in Western blot. Cells in the model group were treated with PA only. After 24 h of drug treatment, each well was stimulated with insulin (100 nM). (**A**) The protein expression and phosphorylation of PI3K/Akt/GSK3β in HepG2 cells. Densitometric analysis of (**B**) P-PI3K/PI3K, (**C**) P-GSK3β/GSK3β, and (**D**) P-AKT/AKT. The results are expressed as the mean ± SD (n = 3). *** *p* < 0.001 vs. control. ^#^ *p* < 0.05, ^##^ *p* < 0.01, ^###^ *p* < 0.001 vs. model. ^&^ *p* < 0.05, compared to TAC.

To determine how TAC promotes glucose absorption, Western blot analysis was performed. As shown in Figure 4, after PA induction, the expression of P-PI3K, P-AKT, and P-GSK3β in cells decreased significantly, but these effects could be notably reversed by TAC, TAC/MSC, or Met treatment (*p* < 0.05). TAC/MSC treatment significantly improved the phosphorylated protein expression of PI3K, Akt, and GSK3β (increased by 40.3 ± 4.0%, 97.4 ± 7.8%, and 57.1 ± 2.3%, respectively, compared to the model group). The results show that MSC could drastically promote TAC’s effect on increasing the expression of phosphorylated proteins of PI3K and Akt (*p* < 0.05), suggesting that TAC/MSC protects the insulin signaling pathway to promote glucose absorption and utilization.

### 2.5. Effects of TAC/MSC on Body Weight, Food Intake, and Fasting Blood Glucose

**Figure 5:**
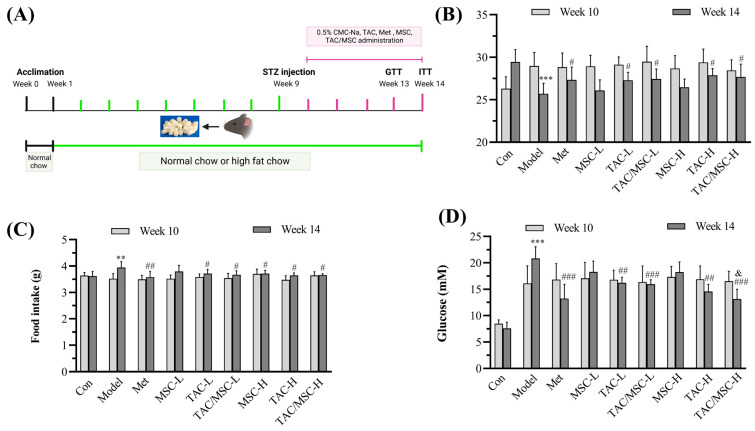
TAC/MSC alleviates STZ and high-fat-diet-induced diabetes in mice. Type 2 diabetic mice were induced by being fed a high-fat diet (HFD) for 8 weeks and then intraperitoneally injected with 100 mg/kg STZ in Week nine. Diabetic mice with hyperglycemia (11 mmol/L or greater) were selected for the experiment. Animals in the model group were given 0.5% CMC-Na, only administered orally. (**A**) C57BL/6J mice were fed a HFD for 14 weeks. (**B**) Body weight, (**C**) food intake, and (**D**) fasting blood glucose levels of mice were monitored in Weeks 10 and 14 at the checkpoint, respectively. Data represent the means ± SD (n = 7). ** *p* < 0.01, *** *p* < 0.001, compared to the control (Week 14). ^#^ *p* < 0.05, ^##^ *p* < 0.01, ^###^ *p* < 0.001, compared to the model (Week 14). ^&^ *p* < 0.05, compared to TAC-H (Week 14).

The protocol of the whole in vivo experimental design is shown in Figure 5A. After STZ treatment, the weight of mice in the model group decreased significantly, while oral feed TAC-L, TAC-H, TAC/MSC-L, and TAC/MSC-H considerably inhibited the weight loss and excessive food intake of mice caused by diabetes (*p* < 0.05) (Figure 5B,C). As reported, mice treated with a high-fat diet with a low dose of STZ exhibited a drastic elevation in fasting blood glucose levels [16]. After four weeks of treatment, the levels of fasting blood glucose in the TAC, TAC/MSC, or Met treatment diabetic group were much lower than those of the diabetic model group, with statistically significant differences (*p* < 0.01; *p* < 0.001; *p* < 0.001, respectively). Furthermore, it is worth noting that MSC-H can significantly promote the hypoglycemic effect of TAC-H (*p* < 0.05) (Figure 5C).

### 2.6. Effects of TAC/MSC on Glucose Tolerance Tests (GTTs) and Insulin Tolerance Tests (ITTs) of T2DM Mice

**Figure 6:**
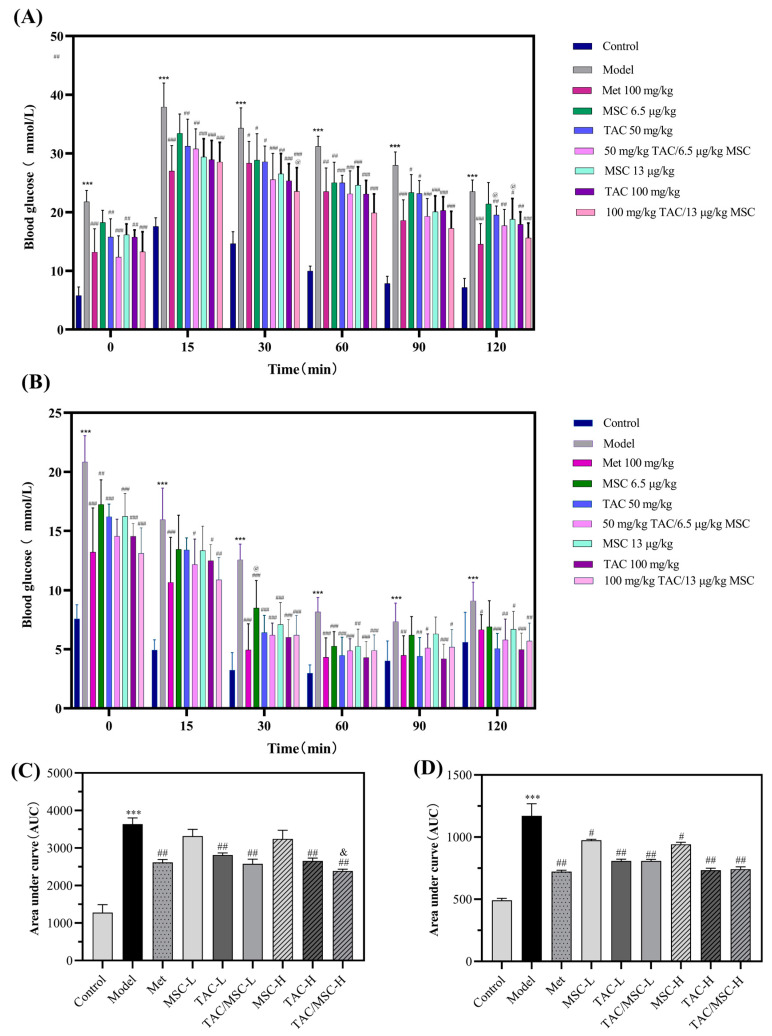
Effects of TAC/MSC on the GTT and ITT of T2DM mice. Animals in the model group were given 0.5% CMC-Na via oral administration only. (**A**) Effects of TAC/MSC on glucose tolerance, (**B**) effects of TAC/MSC on insulin tolerance. (**C**) The areas under the curve (AUCs) of GTT in T2DM mice. (**D**) The AUCs of ITT in T2DM mice. Data represent the means ± SD (n = 7). *** *p* < 0.001 vs. control. ^#^ *p* < 0.05, ^##^ *p* < 0.01, ^###^ *p* < 0.001 vs. model. ^@^ *p* < 0.05 vs. Met group. ^&^ *p* < 0.05 vs. TAC-H.

The experimental results of the GTT are shown in Figure 6A; the blood glucose of mice in each group increased rapidly within 30 min after oral administration of glucose solution. Then, the blood glucose levels of the control group gradually dropped to the initial level within 120 min, while the blood glucose levels of model mice decreased slowly, with the largest AUC (*p* < 0.001; Figure 6C). However, Met and TAC supplementation (50 and 100 mg/kg) reduced blood glucose levels after 60 min, and the AUC of TAC/MSC-H was equivalent to that of the Met group. Compared with TAC-H alone, TAC-H combined with MSC-H achieved a better effect in improving glucose tolerance in mice (*p* < 0.05). The results of the ITT are shown in Figure 6B. The blood glucose of each group of mice decreased rapidly within 0–30 min after insulin injection. Metformin and TAC supplementation significantly improved insulin resistance (*p* < 0.01; Figure 6B,D). MSC also showed some characteristics of improving insulin resistance (*p* < 0.05), but the improvement effect of TAC on insulin resistance was not significantly augmented after being combined with MSC (Figure 6D).

### 2.7. Effects of TAC/MSC on Serum Lipid Metabolism in T2DM Mice

**Figure 7:**
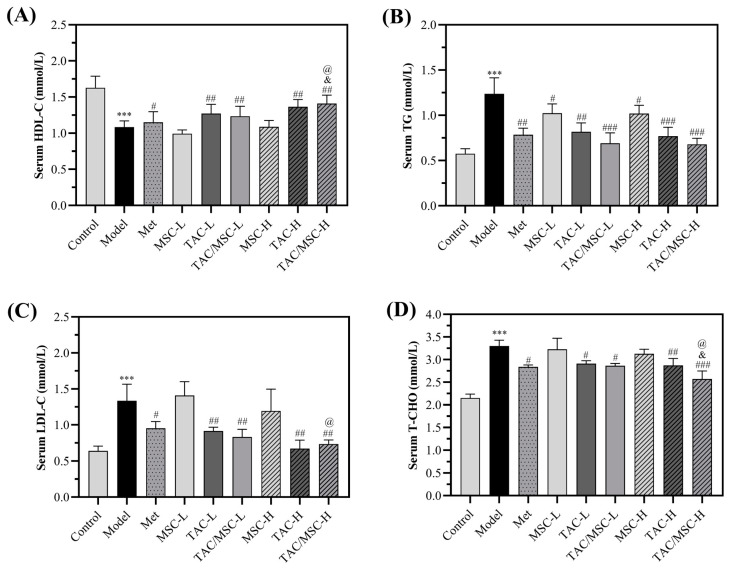
Effect of TAC/MSC on levels of (**A**) high-density lipoprotein cholesterol (HDL-C), (**B**) triglyceride (TG), (**C**) low-density lipoprotein cholesterol (LDL-C), and (**D**) total cholesterol (TCHO) in serum of experimental T2DM mice. Animals in the model group were given 0.5% CMC-Na via oral administration only. Data represent the means ± SD (n = 6). *** *p* < 0.001 vs. control. ^#^ *p* < 0.05, ^##^ *p* < 0.01, ^###^ *p* < 0.001 vs. model. ^@^ *p* < 0.05 vs. Met group. ^&^ *p* < 0.05 vs. TAC-H group.

Compared with the control group, high-density lipoprotein cholesterol (HDL-C, Figure 7A) remarkably declined compared to the control group (*p* < 0.001), and the levels of TG (Figure 7B), total cholesterol (TC, Figure 7D), and low-density lipoprotein cholesterol (LDL-C, Figure 7C) in the serum of model mice significantly increased. Compared with the Met group, considerably increased serum HDL-C content (*p* < 0.05; Figure 7A) and reduced serum LDL-C and T-CHO contents (*p* < 0.05; Figure 7B–D) were observed in the TAC/MSC-H group, proving the superiority of TAC in lowering blood lipids. MSCL and MSCH administration only reduced the TG content in serum but had no noteworthy effect on other lipid components (Figure 7B). The combination of TAC-H and MSC-H treatment extensively reduced the content of TCHO and increased the HDL-C content in serum compared with TAC-H (*p* < 0.05). Interestingly, although MSC itself had little effect on reducing blood lipids, MSC promoted the blood-lipid-lowering effect of TAC.

### 2.8. Effect of TAC/MSC on T2DM-Induced Histopathological Changes in the Liver

**Figure 8:**
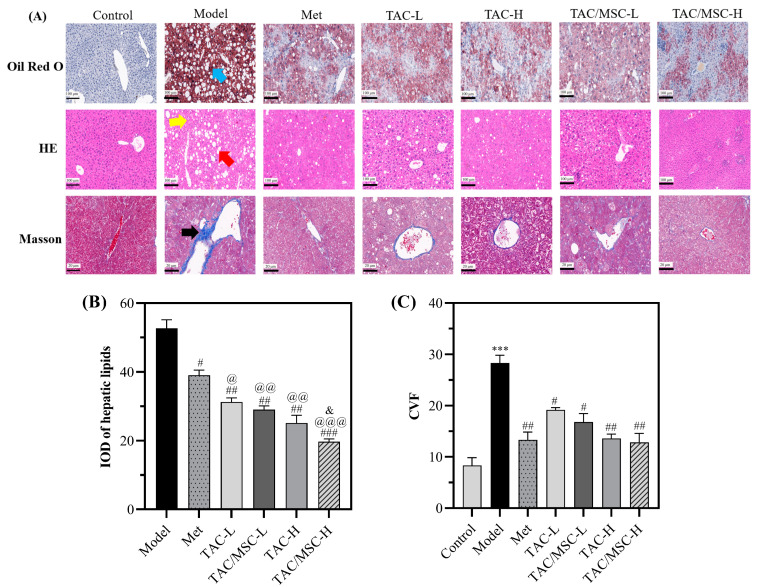
Effects of TAC/MSC on T2DM-induced histopathological changes in the liver. Animals in the model group were given 0.5% CMC-Na via oral administration only. (**A**) Hematoxylin and eosin (H&E) staining, 20×; Oil Red O staining, 20×; and Masson staining, 60×. Liver lipids (blue arrow); focal necrosis (red arrow); mussy hepatic cords (yellow arrow); and LF—liver fibrosis (black arrow). (**B**) Integral optical density (IOD) of the hepatic lipid. (**C**) Collagen volume fraction (CVF). The analysis of IOD via ImageJ software (V1.8.0.112). Data represent the means ± SD (n = 3). *** *p* < 0.001, compared with the control group. ^#^ *p* < 0.05, ^##^ *p* < 0.01, ^###^ *p* < 0.01, compared with the model group. ^@^ *p* < 0.05, ^@@^ *p* < 0.01, ^@@@^ *p* < 0.001 vs. Met group. ^&^ *p* < 0.05 vs. TAC-H.

The structural changes in the liver were examined via H&E staining, Oil Red O staining, and Masson lichun red acid reddish staining (Masson staining) (Figure 8A). The H&E staining showed that hepatocytes in the normal group were closely arranged, while in the model group, liver tissue cells were vacuolated, with white parts accumulated with fat, focal necrosis (red arrow), and messy hepatic cords (yellow arrow). Similarly to the serum lipid detection results (Figure 7), TAC displayed an enhanced effect in reducing liver lipids compared with Met (*p* < 0.05). MSC significantly promoted the lipid-lowering effect of TAC (Figure 8B). Moreover, TAC and TAC/MSC can reduce the collagen volume fraction (black arrow, Figure 8A). The staining results demonstrate the protective effect of TAC on the liver, and the combination of MSC and TAC could better alleviate these pathological changes in the liver.

### 2.9. Effects of TAC/MSC on Proinflammatory Cytokines

**Figure 9:**
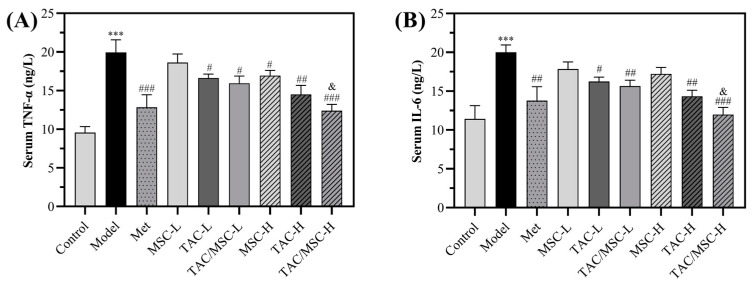
Effects of TAC/MSC on proinflammatory cytokine secretion in serum of T2DM mice. Animals in the model group were given 0.5% CMC-Na via oral administration only. (**A**) TNF-α level, (**B**) IL-6 level in mice serum. IL-6: interleukin-6; TNF-α: tumor necrosis factor alpha. Data represent the means ± SD (n = 5). *** *p* < 0.001, compared with the control group. ^#^ *p* < 0.05, ^##^ *p* < 0.01, ^###^ *p* < 0.01, compared with the model group. ^&^ *p* < 0.05 vs. TAC-H.

As shown in Figure 9, a high-fat diet markedly increased IL-6 and TNF-α levels in mouse serum (an increment of 7.35 ± 1.92 and 8.54 ± 0.85 ng/L, respectively, *p* < 0.001), whereas treatment with TAC dose-dependently decreased their levels. Moreover, the anti-inflammatory effect of TAC/MSC-H was not significantly different from that of the Met group, and there was a substantial difference between the TAC/MSC-H group and TAC group (*p* < 0.05), which proves that MSC could promote a reduction in serum inflammatory factors. In conclusion, the TAC/MSC complex could inhibit the high-fat-diet-induced secretion of proinflammatory cytokines.

### 2.10. Effects of TAC/MSC on Superoxide Dismutase (SOD), Glutathione Peroxidase (GSH-Px), and Catalase (CAT) in Liver

**Figure 10:**
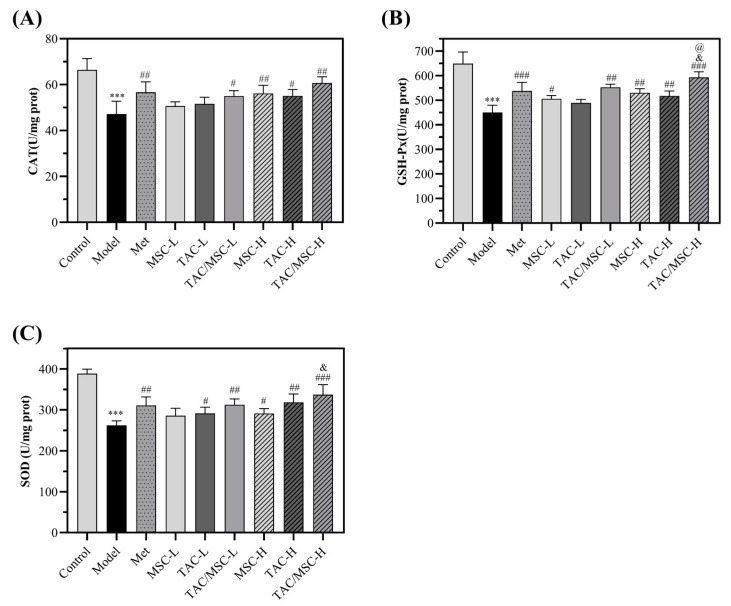
Effects of TAC/MSC on (**A**) CAT, (**B**) GSH-Px, and (**C**) SOD activities in mouse livers. Animals in the model group were given 0.5% CMC-Na via oral administration only. The results are expressed as the means ± SD (n = 5). *** *p* < 0.001 vs. control group. ^#^ *p* < 0.05, ^##^ *p* < 0.01, or ^###^ *p* < 0.01 vs. model group. ^@^ *p* < 0.05 vs. Met group. ^&^ *p* < 0.05 vs. TAC-H. SOD: superoxide dismutase; CAT: catalase; GSH-Px: glutathione peroxidase.

As shown in Figure 10, the amount of CAT, GSH-Px, and SOD decreased extensively in the livers of the diabetic mice compared with the control group (*p* < 0.001). However, the GSH-Px and SOD contents were significantly increased in the TAC/MSC-H (100 mg/kg TAC/13 μg/kg MSC) group compared with the Met group. The results showed that the MSC-H and TAC-H complex could increase the liver antioxidant capacity of diabetic mice.

### 2.11. Effects of TAC/MSC on ROS in the Liver

**Figure 11:**
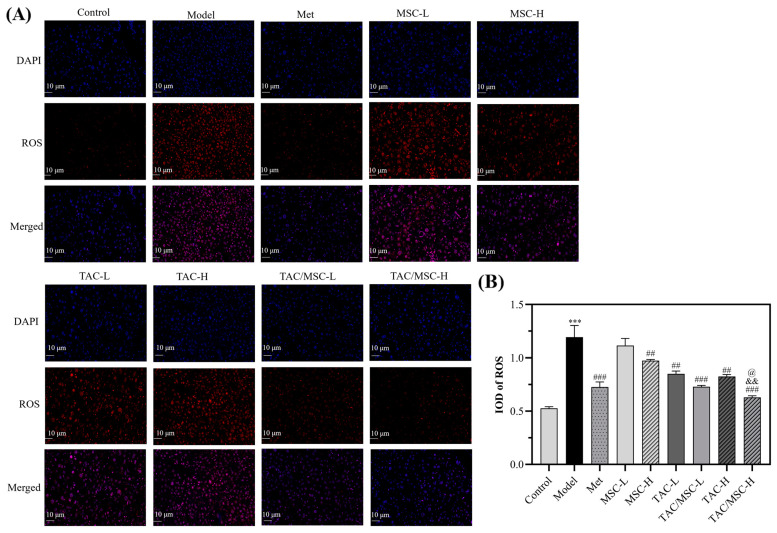
Effect of TAC/MSC on reactive oxygen species (ROS) in the liver of T2DM mice. Animals in the model group were given 0.5% CMC-Na via oral administration only. (**A**) Livers were stained with specific antibodies against ROS (red). Nuclei were stained with 4′,6-diamidino-2-phenylindole (DAPI) (blue). (**B**) The IOD of ROS. The results are expressed as the means ± SD (n = 3). *** *p* < 0.001 vs. control. ^##^ *p* < 0.01, ^###^ *p* < 0.001 vs. model. ^@^ *p* < 0.05 vs. Met group. ^&&^ *p* < 0.01 vs. TAC-H.

To determine the influence of MSC on liver antioxidant capacity, liver ROS was further examined. Figure 11A showed that the number of ROS was significantly increased in the model group. The effect of the TAC/MSC-H group on reducing ROS was considerably improved compared with the Met group (*p* < 0.05; Figure 11B). The ROS results were consistent with the data of liver antioxidant enzymes (Figure 10), and the addition of MSC-H improved the antioxidant capacity of TAC-H (*p* < 0.001; Figure 11B).

### 2.12. Effects of TAC/MSC on P-PI3K, PI3K, P-AKT, AKT, P-GSK3β, and GSK3β in the Liver

**Figure 12:**
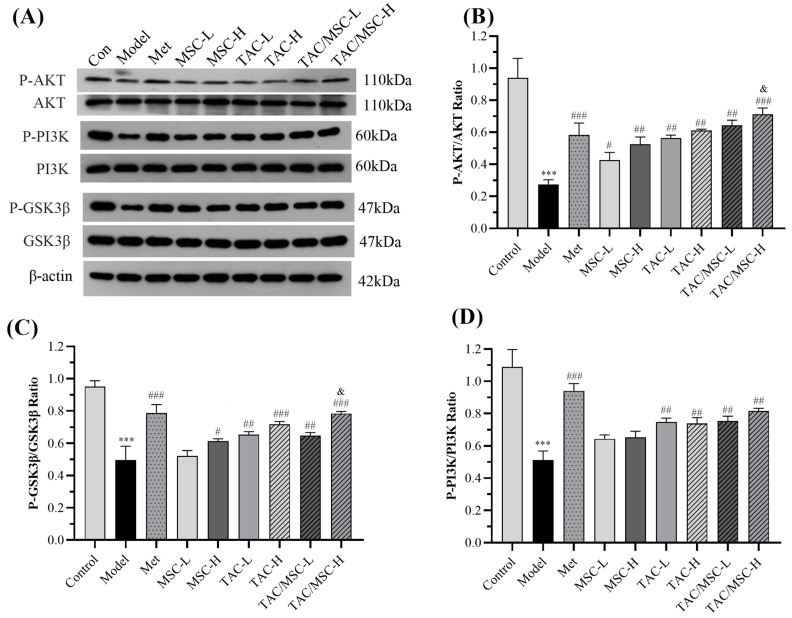
Effect of TAC/MSC on PI3K/AKT/GSK3β signaling pathway in the liver of T2DM mice. Animals in the model group were given 0.5% CMC-Na via oral administration only. (**A**) Protein expression analysis of the livers of experimental mice using P-PI3K, PI3K, P-AKT, AKT, P-GSK3β, and GSK3β antibodies via Western blot. Densitometric analysis of (**B**) P-AKT/AKT, (**C**) P-GSK3β/GSK3β, and (**D**) P-PI3K/PI3K. The results are expressed as the mean ± SD (n = 3). *** *p* < 0.001 vs. control, ^#^ *p* < 0.05, ^##^ *p* < 0.01, ^###^ *p* < 0.001 vs. model. ^&^ *p* < 0.05 vs. TAC-H.

The expression of key proteins of the insulin signaling pathway in T2DM mice was also verified in a similar way. As shown in Figure 12, impaired insulin signaling was observed in the liver of HFD-fed mice, as evidenced by decreased phosphorylation levels of PI3K, Akt, and GSK3β. In contrast, TAC/MSC-H treatment significantly improved the phosphorylated protein levels of PI3K, Akt, and GSK3β compared to the model group (increases of 31.5 ± 7.8%, 43.8 ± 3.8%, and 28.6 ± 7.5%, respectively, *p* < 0.01). No obvious difference in the total protein expression of PI3K, Akt, and GSK3β in the liver was observed among the nine groups. TAC/MSC-H significantly promoted the phosphorylation of AKT and GSK3β compared to the Met group (*p* < 0.05).

### 2.13. Effects of TAC/MSC on SREBP-1c, FAS, ATGL, and HSL in the Liver and White Adipose Tissue (WAT)

**Figure 13:**
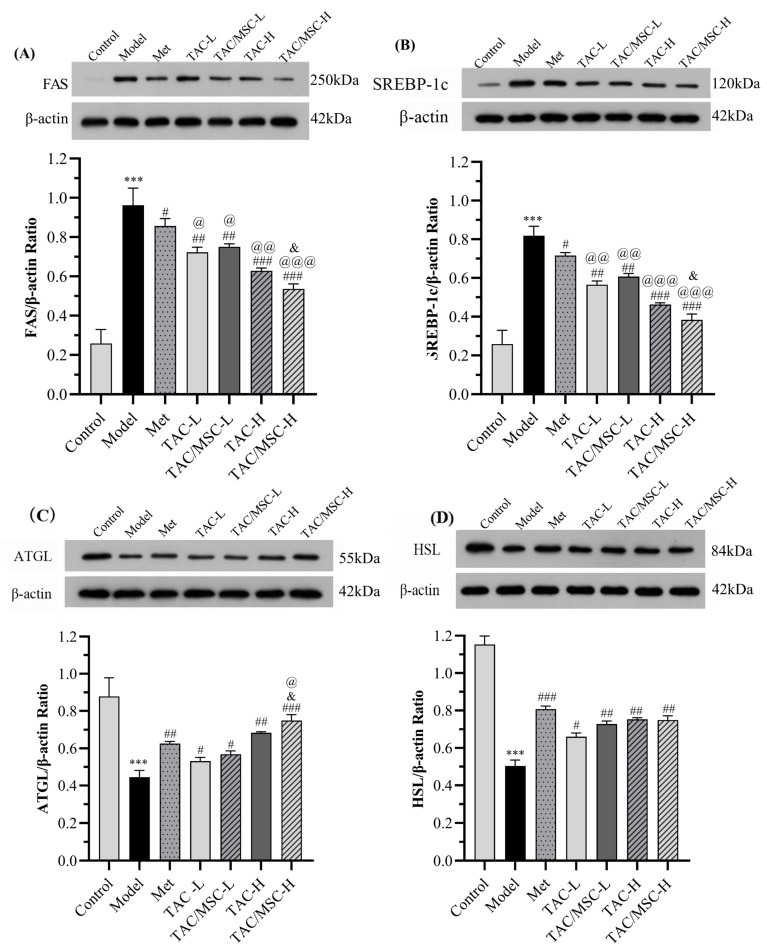
TAC/MSC regulates lipid metabolism in T2DM mice. (**A**,**C**) Protein expression levels of SREBP and FAS in liver tissues (n = 3). (**B**,**D**) Protein expression levels of ATGL and HSL in WAT (n = 3). Animals in the model group were given 0.5% CMC-Na via oral administration only. The results are expressed as the mean ± SD (n = 3). *** *p* < 0.001 vs. control. ^#^ *p* < 0.05, ^##^ *p* < 0.01, ^###^ *p* < 0.001 vs. model. ^@^ *p* < 0.01, ^@@^ *p* < 0.01, ^@@@^ *p* < 0.001 vs. Met group. ^&^ *p* < 0.05 vs. TAC-H.

To further determine whether TAC/MSC can regulate lipid metabolism in mice, the genes involved in lipid metabolism in WAT (epididymal white adipose tissue) and liver tissue were studied. The results show that TAC inhibited the expression of SREBP and FAS in the liver, and there was a remarkable difference compared with the Met group, which proved the superiority of TAC in reducing blood lipids (*p* < 0.05). The significant difference between TAC/MSC-H and TAC-H demonstrates that MSC has a promoting effect on inhibiting the expression of SREBP and FAS in the liver and increasing the expression of ATGL in the WAT of mice (*p* < 0.05) (Figure 13A–C). Although the high-fat diet reduced the expression of ATGL and HSL in the WAT of mice, all of these were effectively reversed via TAC/MSC treatment (Figure 13B–D).

## 3. Discussion

Many studies have confirmed that *C. paliurus* leaves have numerous biological activities, such as hypoglycemic, lipid-lowering, antioxidant, antihypertensive, anticancer, hepatoprotective, and antibacterial activities [23,24,25]. More than ten triterpenoids have been identified from *C. paliurus* [11], and terpenoids have received attention because of their wide range of biological activities. An increasing number of publications have focused on pentacyclic triterpenoids, UA, OA, and BA. These three acids also exist in the triterpene acid of *C. paliurus* and account for a high proportion of triterpenoids in *C. paliurus* [26]. However, the relationship between the contents of UA, OA, and BA in the *Cyclocarya paliurus* triterpene acid complex and its hypoglycemic effect remains unclear. In this study, we optimized the composition of the *Cyclocarya paliurus* triterpene acid complex and found that TAC can improve PA-induced insulin resistance and up-regulate the PI3K/Akt/GSK3β signaling pathway to improve the symptoms of STZ- and high-fat-diet-induced type 2 diabetic mice. Next, we further investigated whether MSC can promote the hypoglycemic effect of TAC. The recommended daily intake of Se for adults according to the Chinese Nutrition Society is 50 μg, and the maximum daily intake is 400 μg. Considering the equivalent dose ratio between humans and animals, the maximum daily Se intake of mice is 52 μg. Therefore, the two doses of MSC utilized in this study, 6.5 and 13 μg/kg, are within the safety range.

Relieving insulin resistance (IR) has been considered the main clinical strategy to improve the metabolism of patients with type 2 diabetes [27]. A large amount of free fatty acids (FFAs), hormones, glycerin, and proinflammatory cytokines are released from adipose tissue and are involved in insulin resistance [28]. Palmitate is one of the most abundant FFAs, accounting for 30–35% of the total FFAs in human plasma [29]. Compared with the TAC group, MSC/TAC co-administration promoted glucose uptake, enhanced glycogen synthesis and reduced gluconeogenesis (Figure 2 and Figure 3). The PI3K/AKT signaling pathway is widely considered to be the most closely related pathway to insulin signal transduction. The activated insulin receptor leads to the phosphorylation of the PI3K p85 subunit, followed by the cascade activation of AKT and the phosphorylation of GSK3β. GSK3β is a major factor regulating glucose metabolism in the liver [9]. We observed that PA-induced insulin-resistant HepG2 cells showed decreased phosphorylation levels of key signaling molecules of the insulin signaling pathway, which is consistent with a previous publication [29]. TAC/MSC co-administration can increase the phosphorylation of AKT, GSK3β, and PI3K (Figure 4). Correspondingly, our animal research results also showed that the dephosphorylation levels of PI3K, AKT, and GSK3β in the livers of HFD-fed mice were significantly increased. In particular, after TAC/MSCH (100 mg/kg TAC/13 μg/kg MSC) administration, the phosphorylation of GSK3β and AKT was remarkably increased compared to that in the TAC-H group, which proves that MSC can promote the TAC effect on improving insulin resistance (Figure 12). Therefore, the present study suggests that the hypoglycemic mechanism of TAC/MSC may be able to improve glucose utilization and promote glycogen synthesis in the body by promoting PI3K/Akt pathway phosphorylation.

A large number of epidemiological studies have shown that the most important risk factor for type 2 diabetes is obesity. Obesity may affect the development of insulin resistance and disease progression [30]. According to the data from the World Health Organization (WHO) in 2011, nearly 90% of diabetes is caused by being overweight. Therefore, we used an 8-week high-fat diet to simulate the process of human obesity. Afterward, a small dose of STZ was injected to partially destroy the function of islet cells, and thus to simulate the damaged state of islet cells in T2DM patients. Figure 4 demonstrates that the T2DM mice induced via a HFD and STZ showed weight loss, a significant increase in blood glucose, and increased food intake. All of the mice in the TAC and TAC/MSC groups had weight gain and decreased food consumption and blood glucose after 4 weeks of administration. We speculated that this change might be related to the improvement in insulin sensitivity (Figure 5).

T2DM induced via HFD feeding in mice is usually accompanied by dyslipidemia, which plays a key role in the pathogenesis of diabetes. Its prominent features include a decrease in HDL-C levels and an increase in TG, TC, and LDL-C levels [31]. Consistent with relevant studies, our results show that the long-term intake of HFD may lead to dyslipidemia. After the administration of TAC, the levels of serum TC, TG, and LDL-C in mice were effectively reduced, and the levels of serum HDL-C were efficiently increased 4 weeks later. TAC/MSC-H can considerably increase HDL-C and reduce LDL-C and T-CHO content compared with Met, which confirms the prospect of the *C. paliurus* triterpene acid complex in reducing blood lipids. Meanwhile, compared with the same dosage of TAC, the combination of TAC and MSC can achieve a better lipid-lowering effect, which confirms the feasibility of the combination of TAC and MSC (Figure 6). We also studied the expression of ATGL and HSL proteins in white adipose tissue and the expression of FAS and SREBP-1c in the liver. ATGL and HSL mainly catalyze the hydrolysis of TG in adipocytes [32]. SREBP-1c regulates the synthesis of fat and cholesterol by regulating the downstream target gene fatty acid synthase (FAS) [33]. The results showed that intragastric TAC/MSC could inhibit the expression of FAS and SREBP protein in mouse liver, and stimulate the expression of ATGL and HSL in mouse WAT, which was consistent with the serum lipid content and liver slices. Compared with *Cyclocarya paliurus* polysaccharide, the *Cyclocarya paliurus* triterpenoid fraction exhibits an excellent effect on reducing blood lipids [34]. In this experiment, TAC and TAC/MSC often had a better effect on reducing blood lipids than Met at the same concentration, which confirms the prospect of the *Cyclocarya paliurus* triterpenoid acid complex as a component of reducing blood lipids in food. In conclusion, the TAC/MSC complex can participate in the regulation of lipid metabolism by increasing the expression of lipid-mobilizing enzymes ATGL and HSL in the white fat of T2DM mice and down-regulating the expression of fatty acid synthase FAS and SREBP-1c in mouse liver.

Oxidative stress also participates in pathophysiological changes in T2DM [35], and hyperglycemia can up-regulate proinflammatory factors and lead to an increased production of reactive oxygen species (ROS), ultimately leading to vascular dysfunction [35]. Se plays a variety of biological functions, such as antioxidation, mainly through selenoproteins [14]. Selenoproteins exert an antioxidant effect through selenoenzymes such as glutathione peroxidases (GPXs). GPXs catalyze the reduction of hydrogen peroxide, lipid peroxide, and phospholipid peroxide into water and alcohol. The results (Figure 10) showed that almost all HFD-fed and STZ-induced T2DM mice had decreased antioxidant enzyme activity. TAC/MSC could increase the content of SOD, GSH-Px, and CAT and reduce ROS in the liver. MSC-H supplementation can reduce liver ROS content, which may be related to the increase in antioxidant enzyme synthesis content in T2DM mice after Se supplementation, and it also confirms that the combination of TAC and MSC can alleviate the symptoms of diabetes by improving the antioxidant capacity of mice.

In summary, the TAC/MSC complex can effectively inhibit the activity of glucose and lipid metabolism enzymes, improve insulin resistance, and alleviate lipid metabolism disorders. These research findings may be a new therapeutic strategy for alleviating oxidative stress and reducing T2DM risks.

## 4. Materials and Methods

### 4.1. α-Amylase Inhibition Assay

α-amylase inhibition assay was carried out via the iodine reaction method described by Kim et al. [36]. In brief, α-amylase (Sigma Aldrich, St. Louis, MO, USA) was dissolved in phosphate-buffered saline (pH 6.8, 0.1 mol/L) at a concentration of 1 unit/mL. PBS was used to dissolve the soluble starch. As the substrate of α-amylase, the concentration of soluble starch is 1% (*w*/*w*). Ten microliters of α-amylase solution (1 unit/mL), 50 µL of test substance, and 290 µL of PBS were mixed at 37 °C for 10 min. After preincubation, 350 µL of 1% soluble starch was added as a substrate, and the reaction was conducted at 37 °C for 30 min. Then, 300 µL of iodine solution (0.1% KI and 0.01% I_2_/0.05 N HCl) was added to the reaction solution, and the absorbance was measured at 620 nm.

### 4.2. Pancreatic Lipase Inhibition Assay

The measurement of pancreatic lipase inhibitory activity using substrate p-nitrophenylbutyrate (PNPB) was carried out as described by Eom et al. but with a slight modification [37]. In short, 30 µL of porcine pancreatic lipase (Sigma Aldrich, St. Louis, MO, USA) was added into 10 mM morpholinepropane sulfonic acid and 1 mM ethylenediaminetetraacetic acid (pH 6.8) with 850 mL Tris buffer solution (100 mM Tris HCl and 5 mM CaCl_2_; pH 7.0) to prepare the enzyme buffer. Then, 100 µL of drug solution was added to 880 µL of enzyme buffer solution and incubated at 37 °C for 15 min. After incubation, 20 µL of substrate solution (10 mM PNPB in dimethyl formamide) was added, the enzyme reaction was allowed to take place at 37 °C for 30 min, and the absorbance was measured at 620 nm. The activity of the negative control was reviewed with and without inhibitors. The inhibitory activity (%) was calculated as follows:

Lipase inhibition (%) = [1 − (A − a)/(B − b)] × 100(1)

where A is the activity of the enzyme with the inhibitor, a is the negative control with the inhibitor, B is the activity of the enzyme without the inhibitor, and b is the negative control without the inhibitor.

### 4.3. Process Optimization

With the inhibition rate of amylase and pancrelipase as the response value, with different concentrations of pentacyclic triterpene acids as the three factors, the optimal action range of each factor was preliminarily determined.

Response surface methodology (RSM) was used to optimize and evaluate the contents of UA, OA, and BA to investigate the effects on the activity of α-amylase and pancreatic lipase in vitro. The effects of the three independent variables A: UA; B: BA; and C: OA on the response (PY) were investigated, and the optimal conditions were checked using the Box–Behnken experimental design of RSM to obtain an optimized prescription compound. Three repetitions of the center operation led to 15 groups of experiments, which optimized the response of each experiment.

Using the program Design Expert V 8.0.6.1, a Box–Behnken design was implemented with three factorial points and three independent variables to determine the proportion of UA, OA, and BA. The experiment was finally replicated under optimum values according to Derringer’s desirable response surface methodology prediction tool for the content of UA, BA, and OA, which could yield the maximum enzyme inhibition rate result.

### 4.4. Cell Culture and Treatments

HepG2 cells were provided by Conservation Genetics CAS Kunming Cell Bank (Kunming, China) and cultured in DMEM with 10% FBS and 1% penicillin/streptomycin at 37 °C with 5% CO_2_ in a humidified atmosphere (Thermo Fisher Scientific, CO_2_ incubator, Waltham, MA, USA). TAC was dissolved in DMSO to obtain a TAC stock solution. To investigate the protective effects and the potential mechanism of TAC, MSC, and the combination of TAC and MSC against palmitic acid (PA)-induced cellular insulin resistance and lipid accumulation, upon reaching 70–80% confluence, cells were treated with 0.1 mM PA with or without TAC (348 μg/L for the content of UA, 354 μg/L for the content of BA, and 398 μg/L for the content of OA), MSC, TAC/MSC, or metformin (10 μg/mL) for 24 h.

### 4.5. MTT Assay

Cells were seeded into a 96-well plate at a density of 1 × 10^4^ cells per well for 24 h and then treated with TAC, MSC, TAC/MSC, or metformin at the indicated concentrations. After the above treatments for 24 h, 20 μL MTT solution (5 mg/mL) was added and incubated for 4 h. Finally, 150 μL DMSO was added into each well, and the absorbance was measured at 490 nm using a microplate reader.

### 4.6. Glucose Consumption Assay

Cells were seeded into a 96-well plate at a density of 8 × 10^3^ cells per well with six wells left as blanks. After reaching 80% confluence, the medium was replaced by DMEM supplemented with 0.1 mM PA and metformin, TAC, MSC, or TAC/MSC at various concentrations for 24 h, and then a glucose assay kit (Nanjing Jian Cheng Bioengineering Research Institute, Nanjing, China) was used to measure the glucose concentration in the medium of each well. Glucose consumption was obtained by subtracting the glucose concentration in the plate well from the glucose concentration of the blank well.

### 4.7. Glycogen Content Assay

Cells were seeded in a 6-well plate at a density of 4 × 10^5^ cells per well and PA, metformin, TAC, and TAC/MSC at various concentrations were used to treat HepG2 cells for 24 h. The glycogen content assay was performed according to the glycogen content test kit (Beijing Solarbio, Shanghai, China) instructions. The absorbance was measured at 620 nm using a microplate reader.

### 4.8. Glucose Production Assay

HepG2 cells were seeded in a 12-well plate (1 × 10^5^ cells per well), and PA, metformin, TAC, and TAC/MSC at various concentrations were used to treat HepG2 cells. After 24 h, the medium was replaced with glucose-free DMEM. After 4 h of incubation, the medium was collected and the glucose concentration was measured using DNS reagent (Yuanye, Shanghai, China). The glucose concentration was normalized to the protein level detected using a BCA Protein Assay Kit (Beyotime, Shanghai, China).

### 4.9. Glucose Production Assay

HepG2 cells were seeded in 6-well plates at a density of 1.5 × 10^5^ cells for 24 h. After 24 h of treatment, cells were washed with PBS, and then lysed on ice for 30 min with cold radio immunoprecipitation assay (RIPA) protein extraction buffer (Beyotime Biotechnology, Beijing, China) containing 1% protease and phosphatase inhibitors. The triglyceride content assay was performed according to the instruction of the TG test kit (Nanjing Jian Cheng Bioengineering Research Institute, Nanjing, China). TG concentrations were standardized to the total protein content measured from the whole cell lysates.

### 4.10. In Vivo Experimental Design

Male SPF-grade C57BL/six mice (16 ± 1 g) were purchased from the Hubei Food and Drug Safety Evaluation Center (Wuhan, China). All animal experiments were carried out in accordance with the Guide for the Care and Use of Laboratory Animals at Huazhong University of Science and Technology. The protocol was approved by the Animal Care Committee of Hubei Province, China (approval number: TY20120158).

After acclimation for one week, 8 mice were randomly selected as the normal group and fed a normal chow diet (5.7% fat, 44.3% carbohydrate, and 19.9% protein (by weight)). The remaining 80 mice were fed an SPF high-fat diet (standard diet 77.5% (*w*/*w*), cholesterol 2%, yolk 10%, lard 10%, bile sodium 0.5%, about 4016 kcal/kg, 30% of calories from fat) as the model group, and then were intraperitoneally (i.p.) injected with 100 mg/kg STZ (dissolved in a pre-chilled 10 *v*/*v* % citrate buffer, pH 4.5) in Week 9. One week later, mice with 8 h fasting blood glucose (FBG) levels in the tail vein higher than 11.1 mmol/L were determined to have type 2 diabetes mellitus (T2DM) and used for further pharmacological studies.

After induction of T2DM, the model group was randomly divided into eight groups (n = 10 per group) as follows: (1) model group (T2DM group) + 0.5% CMC-Na; (2) Met group (T2DM mice fed with 100 mg/kg Met, BW); (3) MSC-L group (T2DM mice fed with 6.5 μg/kg MSC, BW); (4) TAC-L group (T2DM mice fed with 50 mg/kg TAC, BW); (5) TAC/MSC-L group (T2DM mice fed with 50 mg/kg TAC/6.5 μg/kg MSC, BW); (6) MSC-H group (T2DM mice fed with 13 μg/kg MSC, BW); (7) TAC-H group (T2DM mice fed with 100 mg/kg TAC, BW); (8) TAC/MSC-H group (T2DM mice fed with 100 mg/kg TAC/13 μg/kg MSC, BW). All of the samples were orally administered for four weeks. Food intake, weight, and FBG levels were measured every week.

Finally, the mice were sacrificed after anesthesia with 10% chloral hydrate intraperitoneally to collect the liver, white adipose tissues, and blood after overnight fasting (16 h). Serum samples were obtained by centrifuging blood at 4000 rpm/min at 4 °C (YINGTAI TGL16E, Changsha, China). Part of the liver and white adipose tissues were fixed with 10% formalin; other tissues were quickly frozen in liquid nitrogen and then stored in an −80 °C freezer (New Brunswick Science U570-86; Eppendorf, Hamburg, Germany).

### 4.11. Food Intake, Body Weight, Blood Glucose, and Biochemical Analysis

Body weight (g), food intake (g/day), and FBG were recorded within 0 and 4 weeks of administration. Serum TCHO, TG, LDL-C, HDL-C, GSH-Px, and SOD were analyzed using commercial kits (Nanjing Jian Cheng Bioengineering Research Institute, Nanjing, China) according to the manufacturer’s directions.

### 4.12. In Vivo Glucose Tolerance Test (GTT) and Insulin Tolerance Test (ITT)

GTT and ITT were performed to test glucose tolerance and insulin sensitivity using a blood glucometer. After overnight fasting, an i.p. glucose injection (2 g/kg body weight) was performed, and tail vein blood samples were collected at 0, 30, 60, 90, and 120 min after injection. For ITT, insulin was diluted to 0.1 U/mL via 0.9% NaCl. Insulin (1 U/kg) was injected intraperitoneally after 4 h fasting, and tail vein blood samples were collected at 0, 30, 60, 90, and 120 min after injection. The area under the curve (AUC) of glucose against time was calculated to analyze glucose tolerance and insulin tolerance.

### 4.13. Enzyme-Linked Immunosorbent Assay

ELISA was performed according to the Mouse Tumor Necrosis Factor α (TNF-α) ELISA Kit and Mouse Interleukin-6 (IL-6) ELISA Kit instructions (Jiangsu Meimian Industrial Co., Ltd., Yancheng, China). Serum levels of TNF-α and IL-6 were determined at OD 450 nm and corresponded to standard curves.

### 4.14. Histological Assessment

The liver tissue sections were subjected to hematoxylin and eosin (H&E) staining, Masson’s trichrome staining, and Oil Red O staining. Oil Red O, H&E, and Masson’s trichrome staining results of the liver were scanned using a Pannoramic 250/MIDI digital scanner (3D Histech).

### 4.15. Immunofluorescence Analyses

Liver sections were incubated with 10% goat serum at 4 °C overnight to block non-specific expression. A primary antibody for ROS was used to incubate it with liver slices in 2% serum overnight at 4 °C. The next day, the slices were washed with PBS for 5 min three times and then incubated in rabbit antigoat IgG diluent at 25 °C for 1 h. Then, the slices were washed again with PBS for 5 min three times and stained with DAPI. Staining results of the liver were scanned using a Pannoramic 250/MIDI digital scanner (3D Histech, Budapest, Hungary).

### 4.16. Western Blot

Liver samples or cells were lysed in RIPA buffer (Servicebio, Wuhan, China). The proteins were then separated via centrifugation at 12,000 rpm for 10 min. Protein concentration was determined using the BCA Protein Assay Kit (Beyotime, Shanghai, China). Equal amounts of proteins were separated via 10% sodium dodecyl sulfate-polyacrylamide gel electrophoresis (SDS-PAGE) and transferred to polyvinylidene fluoride (PVDF) membranes (Millipore, Billerica, MA, USA). After being blocked with 5% non-fat milk at room temperature for 30 min, the samples were then incubated with primary antibodies overnight at 4 °C. Then, the membranes were incubated with secondary antibodies at room temperature for 1 h. The bands were detected using an enhanced chemiluminescence (ECL) kit, and blots were quantified via IPP 6.0 software.

### 4.17. Statistical Analysis

All results are shown as the mean and standard deviation from at least three independent experiments. Statistical analyses were performed with one-way ANOVA. *p* values < 0.05 were considered to be statistically significant.

## Figures and Tables

**Table 1:** The Box–Behnken response surface design and corresponding response values.

RUN	UA (μg/mL)	BA (μg/mL)	OA (μg/mL)	Pancreatic Lipase Inhibition (%)	α-Amylase Inhibition (%)
1	100	100	250	45.38	34.48
2	400	100	250	43.8	43.95
3	100	400	250	42.83	48.79
4	400	400	250	56.52	59.74
5	100	250	100	60.51	46.51
6	400	250	100	61.08	54.47
7	100	250	400	62.56	53.48
8	400	250	400	63.18	59.49
9	250	100	100	58.08	44.47
10	250	400	100	60.93	59.01
11	250	100	400	58.2	46.84
12	250	400	400	61.9	62.31
13	250	250	250	62.03	58.98
14	250	250	250	64.51	52.71
15	250	250	250	62.5	55.62

**Table 2:** Analysis of variance of the compound with the pancreatic lipase inhibition (%) as the response value.

Source	Sum of Squares	Df	Mean Square	F Value	*p*-Value Prob > F	
Model	781.98	9	86.89	17.77	0.0005	**Significant**
A	56.6	1	56.60	11.58	0.0114	
B	53.58	1	53.38	10.96	0.0129	
C	10.75	1	10.75	2.20	0.1817	
AB	92.82	1	92.82	18.98	0.0033	
AC	3.88	1	3.88	0.79	0.4025	
BC	0.19	1	0.19	0.039	0.8498	
A^2^	204.25	1	204.25	41.77	0.0003	
B^2^	270.88	1	270.88	55.4	0.0001	
C^2^	92.44	1	92.44	18.91	0.0034	
Residual	34.23	7	4.89			
Lack of Fit	28.08	3	9.43	6.34	0.0532	**Not significant**
Pure Error	5.95	4	1.49			
Cor Total	816.21	16				

**Table 3:** Analysis of variance of the compound with α-amylase inhibition (%) as the response value.

Source	Sum of Squares	Df	Mean Square	F Value	*p*-Value Prob > F	
Model	808.70	9	88.96	18.34	0.0005	**Significant**
A	147.79	1	147.79	30.17	0.0009	
B	451.44	1	451.44	92.14	<0.0001	
C	39.08	1	39.08	7.98	0.0256	
AB	0.55	1	0.55	0.11	0.7475	
AC	0.95	1	0.95	0.19	0.6722	
BC	0.23	1	0.23	0.047	0.8353	
A^2^	66.16	1	66.16	13.5	0.0079	
B^2^	77.82	1	77.82	15.89	0.0053	
C^2^	25.18	1	25.18	5.14	0.0577	
Residual	34.29	7	4.9			
Lack of Fit	10.15	3	3.38	0.56	0.6668	**Not significant**
Pure Error	24.14	4	6.04			
Cor Total	843	16				

## Data Availability

Not applicable.

## Abbreviations

TAC, *Cyclocarya paliuru* triterpenoid acid complex; MSC, Se-methylselenocysteine; UA, ursolic acid; OA, oleanolic acid; BA, betulinic acid; TAs, triterpenoid acids; Met, metformin; PBS, phosphate-buffered saline; PNPB, p-nitrophenylbutyrate; RIPA, radio immunoprecipitation assay; HFD, high-fat diet; STZ, streptozotocin; T2DM, type 2 diabetes mellitus; SOD, superoxide dismutase; GPx, glutathione peroxidase; CAT, catalase; PI3K, phosphoinositide 3-kinase; AKT, protein kinase B; GSK3β, glycogen synthase kinase 3 beta; TCHO, total cholesterol; TG, triglyceride; LDL-C, low-density lipoprotein; HDL-C, high-density lipoprotein; GTT, glucose tolerance test; ITT, insulin tolerance test; AUC, area under the curve; H&E staining, hematoxylin and eosin staining; ROS, reactive oxygen species; HSL, hormone-sensitive lipase; ATGL, adipose triglyceride lipase; WAT, white adipose tissue; SREBP, sterol regulatory element-binding protein; FAS, fatty acid synthase.
